# Gout Arthritis During Admission for Decompensated Heart Failure—A Descriptive Analysis of Risk Factors, Treatment and Prognosis

**DOI:** 10.3389/fmed.2022.789414

**Published:** 2022-02-14

**Authors:** Fabian Ritter, Fabian Franzeck, Julian Geisshardt, Ulrich A. Walker, Michael Osthoff

**Affiliations:** ^1^Division of Internal Medicine, University Hospital Basel, Basel, Switzerland; ^2^Department of Research and Analytical Services, University Hospital Basel, Basel, Switzerland; ^3^Department of Rheumatology, University Hospital Basel, Basel, Switzerland; ^4^Department of Clinical Research, University of Basel, Basel, Switzerland

**Keywords:** decompensated heart failure, gout, arthritis, hyperuricemia, inflammation

## Abstract

**Background:**

Chronic heart failure and hospital admissions are well-known risk factors for acute gouty arthritis. However, in-depth analyses of patients admitted for decompensated heart failure (DHF) who subsequently developed a gout attack are sparse. This study aims to characterize DHF patients who developed a gout attack during their inpatient treatment and describe potential risk factors, its consequences, and its management in the setting of heart failure exacerbation.

**Methods:**

Retrospective chart review of 50 patients with an admission diagnosis of DHF who subsequently experienced a gout attack during admission at a Swiss tertiary care hospital between 2018 and 2020. Patients with a refusal of the general research consent were excluded (*n* = 10).

**Results:**

A gout attack developed in 66/1,832 (3.6%) DHF admissions of whom 50 individual patients were analyzed. Patients were predominately male (76%), of advanced age (median 80.5 years), with several comorbidities including chronic kidney disease (74%), comorbid gout (70%, only 43% on urate lowering therapy) and hyperuricemia (median 547 μmol/l, IQR 434–667 μmol/l). Diuretics were intensified in all patients. Acute gout presented as polyarticular arthritis (62%) and was often accompanied by fever (30%). Joint aspiration was performed in 32%, and intra-articular steroid injections administered in 20% of patients. Median length of stay and 6-month mortality were 16 days (IQR 12–25) and 32%, respectively, compared to 9 days (IQR 6–14) and 16% for DHF patients without a gout attack.

**Conclusion:**

Our study highlights features of gout attacks in the context of DHF including the absence of comorbid gout in a significant proportion of patients, the presence of polyarticular disease during the flare, and a poor prognosis. The present study identifies the necessity to better address gout as a comorbidity in DHF patients and may assist clinicians in identifying DHF patients at risk for a gout attack.

## Introduction

Gout is one of the most common inflammatory joint diseases and strongly associated with multimorbidity (1). The prevalence of gout increases with age (2). Recent studies have indicated that gouty arthritis is a frequent and disabling complication of heart failure, another prevalent condition in elderly patients (3). The main risk factor for monosodium urate (MSU) crystal deposition and subsequent development of gout is hyperuricemia. Several factors that aggravate hyperuricemia, such as certain medical conditions or drugs are associated with the risk of developing acute gout (4).

Chronic kidney disease and diuretic use can contribute to decreased renal excretion of urate and may thereby result in hyperuricemia (5, 6). Thus, heart failure patients appear to be at higher risk of hyperuricemia due to the frequent presence of concomitant renal failure and the frequent use of diuretics. In addition, sodium retention in the context of decompensated heart failure (DHF) may stimulate the renal urate anion exchanger URAT1 (7). Admission for DHF is related to volume overload and congestion in the majority of patients and higher than baseline doses of diuretics are usually administered. As a result, this population may have an increased risk for inpatient acute gout episodes.

However, many factors are associated with an inpatient gout attack, which is described as a multifactorial event influenced by gout and hospital-related factors (8). An analysis of patients hospitalized in New Zealand revealed nine predictors of an inpatient flare, including pre-admission urate >360 μmol/l, acute kidney injury, surgery or adjustment of urate lowering therapy and diuretics prior to flare (9). However, this prediction model was developed for patients with a known history of gout and a variety of admission diagnoses.

Gout attacks occurring during hospitalization cause additional morbidity, such as severe pain and immobility, and may prolong the length of stay (10). Even though acute gout in patients admitted for DHF presents a distinct clinical problem, it has not yet been thoroughly studied. A North American case series reported an incidence of 3.3% (9 out of 271) of gout attacks from intensified diuretic use in patients hospitalized with a primary admission diagnosis of heart failure. In their study they eventually characterized 27 DHF patients and suggested an important association between increased diuretic use and subsequent gout attacks (11).

Given the sparse evidence regarding the factors associated with gouty arthritis in heart failure patients, the aim of the present study is to provide a descriptive analysis of patients hospitalized for decompensated heart failure who subsequently developed an acute gout attack during hospitalization and evaluate potential risk factors, its consequences and management in the setting of heart failure exacerbation.

## Materials and Methods

### Study Population

We performed a retrospective chart review of patients admitted to the University Hospital Basel, for DHF, between January 1, 2018, and March 31, 2020. The University Hospital Basel is a 770-bed tertiary care hospital in Switzerland serving a catchment area of ~1 million inhabitants and the primary referral hospital for complex cardiology and rheumatology patients in Northwestern Switzerland. To be eligible for the primary analysis, DHF patients had to have developed a gout attack during the same admission. The above mentioned period was chosen according to a brief ICD-10 code query in order to being able to analyse ~60 DHF patients with a gout attack during admission. The Ethics Committee of Northwestern and Central Switzerland approved the study (EKNZ 2020-00547) with a waiver for informed consent. However, patients were excluded from the analysis if the general research consent of the University Hospital Basel for the use of routinely obtained personal and medical data had been previously declined.

To identify eligible patients, several strategies were applied. First, we performed an ICD-10 code search of discharge summaries using heart failure- and gout-specific codes for primary and secondary diagnosis (ICD-10 I11.00, I11.01, I13.20, I13.21, I50.x; ICD-10 M10.x). The second strategy involved a cross referenced 'text search' using structured query language to search for specific words within the diagnosis lists in the electronic hospital records (Search terms for the DHF diagnosis included “heart failure” OR “cardiac” AND “decompens” OR “acute.” For the gout diagnosis search terms included “gout” OR “podagra” OR “gonagra”).

To maximize the identification of all relevant cases, we also searched the rheumatology consultation records regarding gout flares and inpatient records of synovial fluid showing monosodium urate crystals. Lastly, we also identified subjects with an acute gout episode through a prescription search of the anti-gout drug colchicine.

The diagnosis of both, DHF and acute gout, was subsequently confirmed by hospital records review. Inclusion criteria for DHF patients consisted of all patients diagnosed with DHF (*de novo* diagnosis or exacerbation of a known heart failure) at the first day of admission. A gout attack was defined as a new episode of one or several painful, swollen, or erythematous joints during admission judged to be an acute gout attack by the attending physician or consultant rheumatologist. The acute gout attack was confirmed either by positive monosodium urate crystals in the joint fluid analysis or by fulfilling the 2015 American College of Rheumatology/European League Against Rheumatism (ACR/EULAR) gout classification criteria (12). Hence, we reduced the possibility that individuals with conditions which often mimic the natural course of gout attacks (e.g., pseudogout) could have been mistakenly included as having an acute gout episode.

Patients were excluded if symptoms of an acute gout flare were already present at the time of admission or if they had any surgical procedure during the same admission prior to the acute gout attack. For patients with more than one admission during the study period, only data from the first admission were analyzed. Additionally, only the first gout attack per hospitalization was considered, if there were multiple flares.

Subsequently, we also analyzed the original DHF population generated by the ICD-10 code search of discharge summaries using heart failure-specific codes for primary and secondary diagnosis and including patients with a secondary diagnosis of heart failure who were additionally identified by the text search query. However, diagnosis of DHF was not verified by chart review in this entire DHF cohort, and parameters requiring manual extractions (such as comorbidities, drugs, etc.) were not analyzed. Hence, patients who were not captured by ICD coding or text search, may have been missed. On the other hand, some patients may have developed DHF only during hospitalization. Due to these limitations, we elected to only report key characteristics of the entire DHF cohort.

### Data Collection and Statistics

We recorded data on demographic parameters, clinical and laboratory characteristics, concomitant medications, on how the diagnosis of gout was established and about the treatment of DHF and the acute gout attack. The degree of comorbidities was assessed by the age-adjusted Charlson Comorbidity Index, a score that ranges from 0 to more than 20 points (13).

Data on demographics and laboratory results were electronically extracted from the hospital data management system. The laboratory data collected included the first laboratory value within 24 h after admission, the peak value prior to the acute gout attack (from admission until 1 day before the date of gout symptom onset) and the peak value at the start of the gout episode (+/−2 days). All clinical data were manually extracted from the patients' electronic clinical records. Data were collected and managed using REDCap electronic data capture tools and analyzed using descriptive and summary statistics. Continuous variables were reported as median [interquartile range (IQR)] and were compared using non-parametric tests (Mann-Whitney U and Wilcoxon test for unpaired and paired observations, respectively), if not normally distributed or as mean +/–SD and were compared using the Student's *t*-test. Categorical variables were expressed as proportions and counts and compared using the Fisher's exact test. Tests were done at the 2-sided 5% significance level. All analyses were performed with the use of SPSS version 22 software (IBM, Chicago, Illinois).

## Results

### Cohort

**Figure 1:**
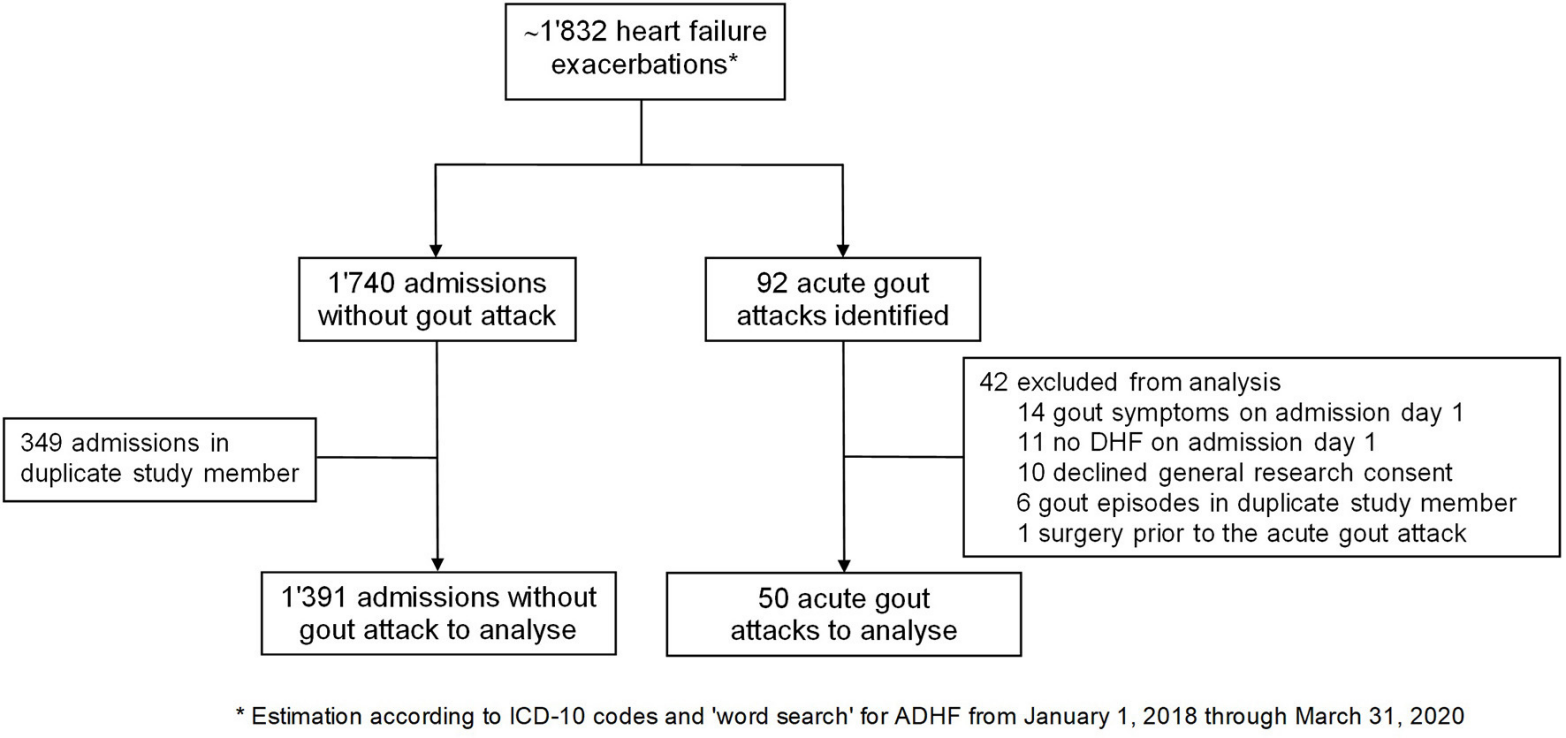
Flowchart of patient selection. ADHF, acute decompensated heart failure; ICD, International Statistical Classification.

Between January 2018 and March 2020 ~1'832 DHF admissions occured at our center. We subsequently identified a total of 92 acute gout attacks in this population (5.0%). However, 14 patients were excluded as they had concomitant heart failure and gout symptoms on admission day one. Notably, in 4 of these, intensification of diuretics was administered prior to the hospitalization. Another 11 patients were excluded because heart failure symptoms developed during hospitalization but were not present at the time of admission. One patient was excluded due to a surgical intervention prior to the acute gout attack. In total, 66 hospitalizations fulfilled the criteria of DHF on admission and developing an acute gout episode during hospitalization. We therefore estimated the incidence of gout flares in patients admitted for DHF to be 3.6% (95% confidence interval 2.8–4.6%). After exclusion of patients who declined the general research consent and patients with more than one admission during the study period, a total of 50 individual DHF patients who developed gout during hospitalization (= gDHF patients) were included in our analysis (Figure 1). On the other hand, 1,391 patients were admitted at least once with DHF and did not suffer from a subsequent gout attack (= nDHF patients).

Gout DHF patients were predominantly male (76%) with a median age of 80.5 years (IQR 75–84). The median hospital length of stay (LOS) in these patients was 16 days (IQR 12–25). Most patients had a poor NYHA functional status on admission (88% NYHA III or IV). The most common triggers for acute decompensation to be reported in this cohort were infection (19/50 of patients) and/or cardiac arrhythmia (15/50 of patients). Non-gout DHF patients were of similar age (median 79 years), but gender was almost equally distributed (54% male) and they had a markedly shorter LOS (median 9 days, IQR 6–14). Baseline characteristics of the cohort are shown in Table 1.

**Table 1:** Characteristics of the patient cohort.

	**gDHF (***n*** = 50)**	**nDHF (***n*** = 1,391)**
Age in years, median (IQR)	80.5 (75–84)	79 (72–86)
Male sex, number (%)	38 (76)	746 (54)
**NYHA class, number (%)**		
II	3 (6)	
III/IV	44 (88)	
LVEF in %, median (IQR)	45 (33–59)	
<40	19 (44)	
40–49	5 (12)	
≥50	19 (44)	
BMI* in kg/m^2^, median (IQR)	26.2 (23.9–30.1)	
Cause of chronic heart failure**, number (%)		
Hypertension	31 (62)	
Coronary artery disease	31 (62)	
Cardiac arrhythmia	30 (60)	
Valvular heart disease	28 (56)	
Cardiomyopathy	3 (6)	
**Comorbidities, number (%)**		
Hypertension	47 (94)	
CKD	37 (74)	
Stage 2 (eGFR 60–89 ml/min)	2 (5)	
Stage 3 (eGFR 30–59 ml/min)	31 (84)	
Stage 4 (eGFR 15–29 ml/min)	4 (11)	
Anemia	37 (74)	
Cardiovascular disease	35 (70)	
History of gout	35 (70)	
Acute kidney injury	29 (58)	
Diabetes mellitus	28 (56)	
Dyslipidemia	25 (50)	
COPD	21 (42)	
Active alcohol drinking	8 (16)	
OSAS	7 (14)	
Cancer	6 (12)	
Urinary tract stones	3 (6)	
Age-adjusted Charlson Comorbidity Index (12), median (IQR)	8 (7–10)	7 (5–8) *n* = 1'295
**Medication on admission, number (%)**		
Any diuretic	47 (94)	
Loop diuretic	43 (86)	
Thiazides	11 (22)	
Aldosterone antagonist	12 (24)	
Beta-Blocker	39 (78)	
ACE inhibitor	16 (32)	
ARB	16 (32)	
Losartan	3 (19)	
Sacubitril–valsartan	1 (2)	
Calcium channel blockers	12 (24)	
Antihyperlipidemic drug	30 (60)	
Antidiabetic drug	22 (44)	
SGLT-2i	2 (9)	
Low-dose aspirin	15 (30)	

Data are number of patients (%) or median (IQR). gDHF, decompensated heart failure patients who developed gout during hospitalization; nDHF, decompensated heart failure patients who did not suffer from a subsequent gout attack; IQR, interquartile range; NYHA, New York Heart Association; LVEF, left ventricular ejection fraction; BMI, body mass index; CKD, chronic kidney disease; COPD, chronic obstructive pulmonary disease; OSAS, obstructive sleep apnea syndrome; ACE, angiotensin-converting enzyme; ARB, angiotensin-receptor blocker; SGLT-2i, sodium-glucose co-transporters-2 inhibitors. Data missing for LVEF for 7 patients. For 3 patients NYHA status was missing. *BMI was calculated according to minimal weight during admission. **Percentage adds up to more than 100% as in some patients multiple causes for chronic heart failure were stated.

Comorbidities such as hypertension (94%), anemia (74%), cardiovascular disease (70%) and diabetes mellitus (56%) were encountered frequently in gDHF patients, which is illustrated by a high age-adjusted Charlson Comorbidity Index [median 8 (IQR 7–10) vs. 7 (IQR 5–8) for nDHF patients]. In particular, acute and stage 3 chronic renal failure [according to the National Kidney Foundation–Kidney Disease Outcomes Quality Initiative (NKF-KDOQI) guideline (14)] was diagnosed in 29 (58%) and 31 (62%) gDHF patients, respectively. A history of gout was mentioned in 35/50 (70%) patients, of whom only 15/35 (43%) patients were on any urate lowering therapy (ULT) at the time of admission. Prior to admission, almost all gDHF patients were treated with diuretics (94%).

### Laboratory Data

On admission, gDHF patients had a markedly elevated median NT-proBNP concentration of 7'749 ng/l (IQR 3'720–13'644). In addition, moderate to severe renal impairment was present in most gDHF patients (median eGFR 36 ml/min; IQR 24–43 ml/min). Serum uric acid levels were considerably elevated with a median serum urate level of 547 μmol/l (IQR 429–668 μmol/l). Notably, of those patients on ULT, only three patients (20%) had achieved the EULAR recommended serum urate target levels of <360 μmol/l for patients without tophaceous gout (15). In contrast, nDHF patients had a preserved renal function [median eGFR 52 ml/min (IQR 34–71 ml/min)] and lower serum uric acid levels on admission [median 414 μmol/l (IQR 321–527 μmol/l)].

**Figure 2:**
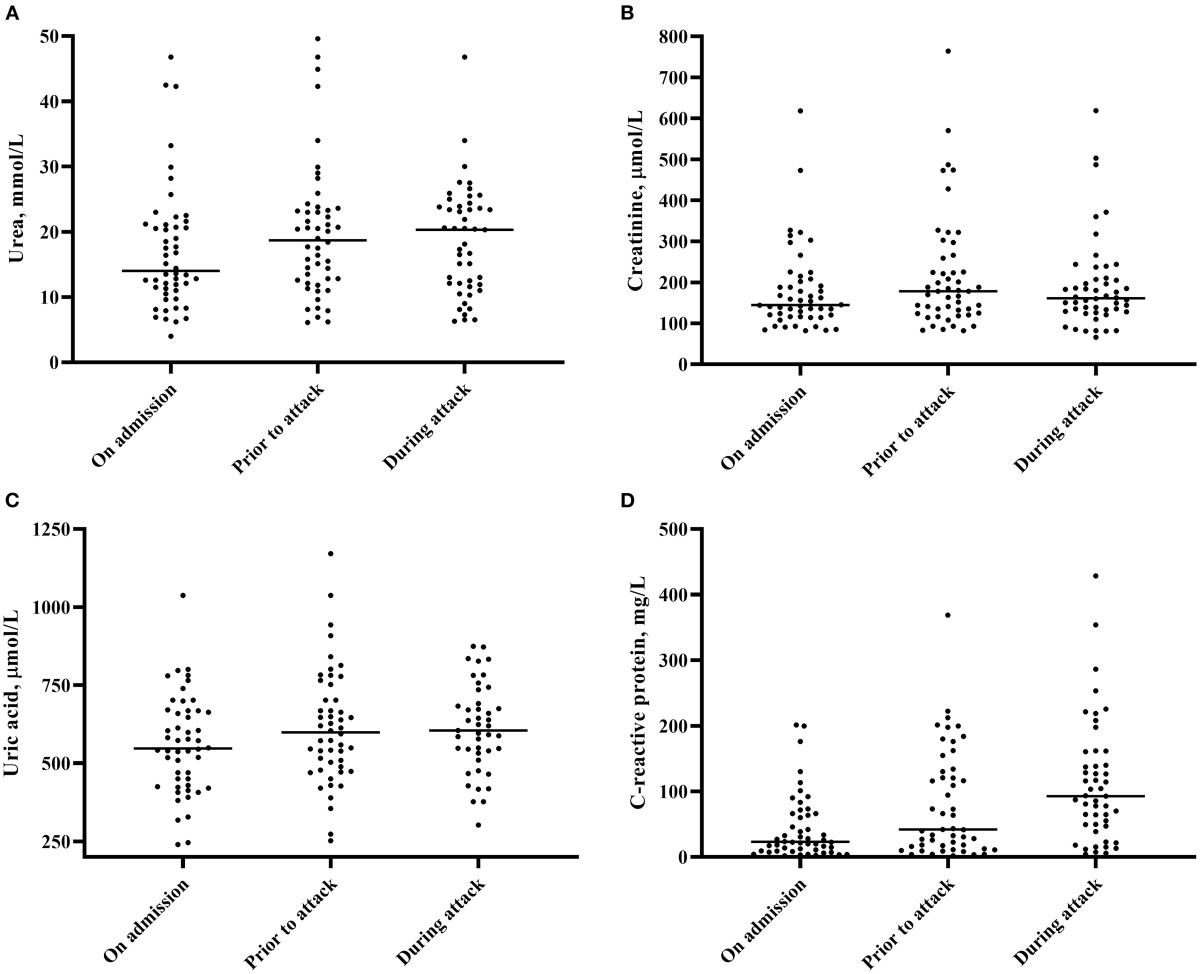
Changes in serum urea **(A)**, serum creatinine **(B)**, serum uric acid **(C)** and serum C-reactive protein concentration **(D)**. Medians are depicted.

A non-significant decrease in renal function [median estimated glomerular filtration rate (eGFR) on admission 35.5 ml/min vs. 30.5 ml/min before the gout attack, *p* = 0.42] and a gradual increase in serum uric concentration (median 547 μmol/l on admission vs. 605 μmol/l before the gout attack, *p* = 0.079) was observed in gDHF patients during the admission leading up to the onset of acute gouty arthritis (Figure 2). In line with the acute phase reaction during the gout attack, the median C-reactive protein (CRP) levels gradually increased toward the acute gout episode (Figure 2, *p* < 0.001).

### Hospital Treatment Prior to the Acute Gout Attack

Prior to the acute gout attack 15/50 (30%) gDHF patients were admitted to an intensive or intermediate care unit compared to 351/1,391 (25%) nDHF patients during their entire hospital stay. Increased dosages of diuretics were administered in all gDHF patients, including intravenous loop diuretics (98%), increased oral dosages of loop diuretics (42%), sequential nephron blockade with thiazides (32%) and aldosterone antagonists (24%) prior to the gout attack. As a consequence of increased diuretic use, the median body weight change during admission in these patients was −6.3 kg (IQR, −3.1 to −8.9). ULT had been discontinued in 8/15 (53%) of patients prior to the onset of acute gouty arthritis.

### Diagnosis and Management of Acute Gout

The median time from admission to onset of arthritis symptoms was 6 days (IQR 3–10 days). Oligo- or polyarticular arthritis was observed in 31 patients (62%). The classic presentation of an acute arthritis with involvement of the metatarsophalangeal joint of the great toe was encountered in only 14 patients (28%). In polyarticular presentation, the most frequently affected joints included wrist or finger joints in 16 patients (52%). In contrast, knee involvement was the most prevalent monoarticular arthritis (7/19, 37%). During the acute gout episode, fever (38°C or greater) was present in 15 patients (30%) with temperatures ranging from 38.0 to 39.2°C. Fever was more prevalent in oligo- or polyarticular (42%) than monoarticular (11%) arthritis (*p* = 0.026).

Diagnosis of gout was confirmed by joint fluid analysis in 16/50 (32%) patients. A rheumatologist was consulted in the majority of patients (54%).

**Figure 3:**
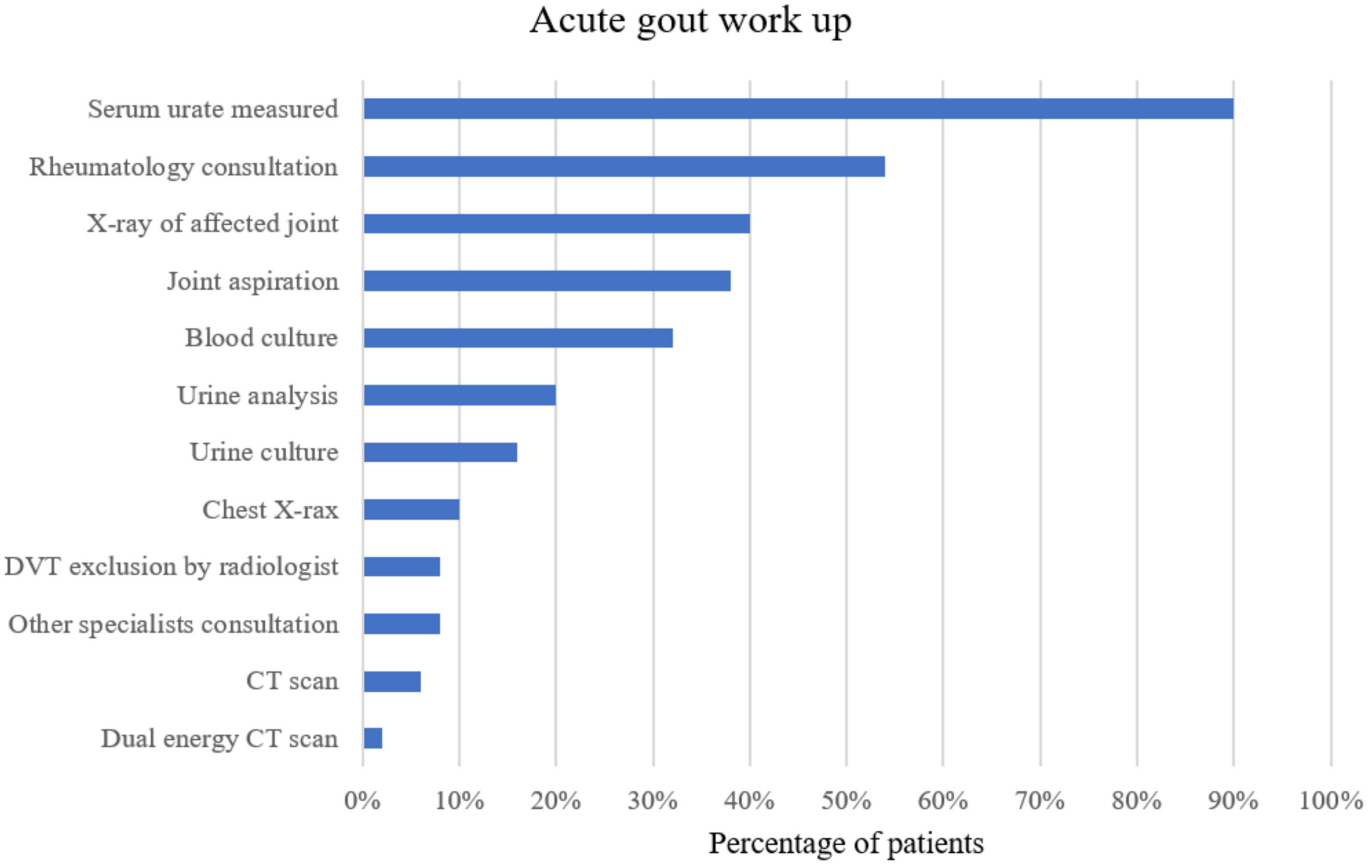
Work-up for acute gout attack in percentage of patients with at least one of the investigations mentioned above. DVT, deep vein thrombosis; CT, computed tomography.

Before the diagnosis of an acute gout attack was established, a number of investigations were performed in several patients. Particularly, work-up for infectious diseases was frequently done in patients with fever and a high CRP. For example, in patients with febrile temperatures additional blood cultures (13/15, 87%), urine cultures (6/15, 40%) or a chest X-ray (4/15, 27%) were obtained. A complete list of additional investigations performed is summarized in Figure 3.

**Figure 4:**
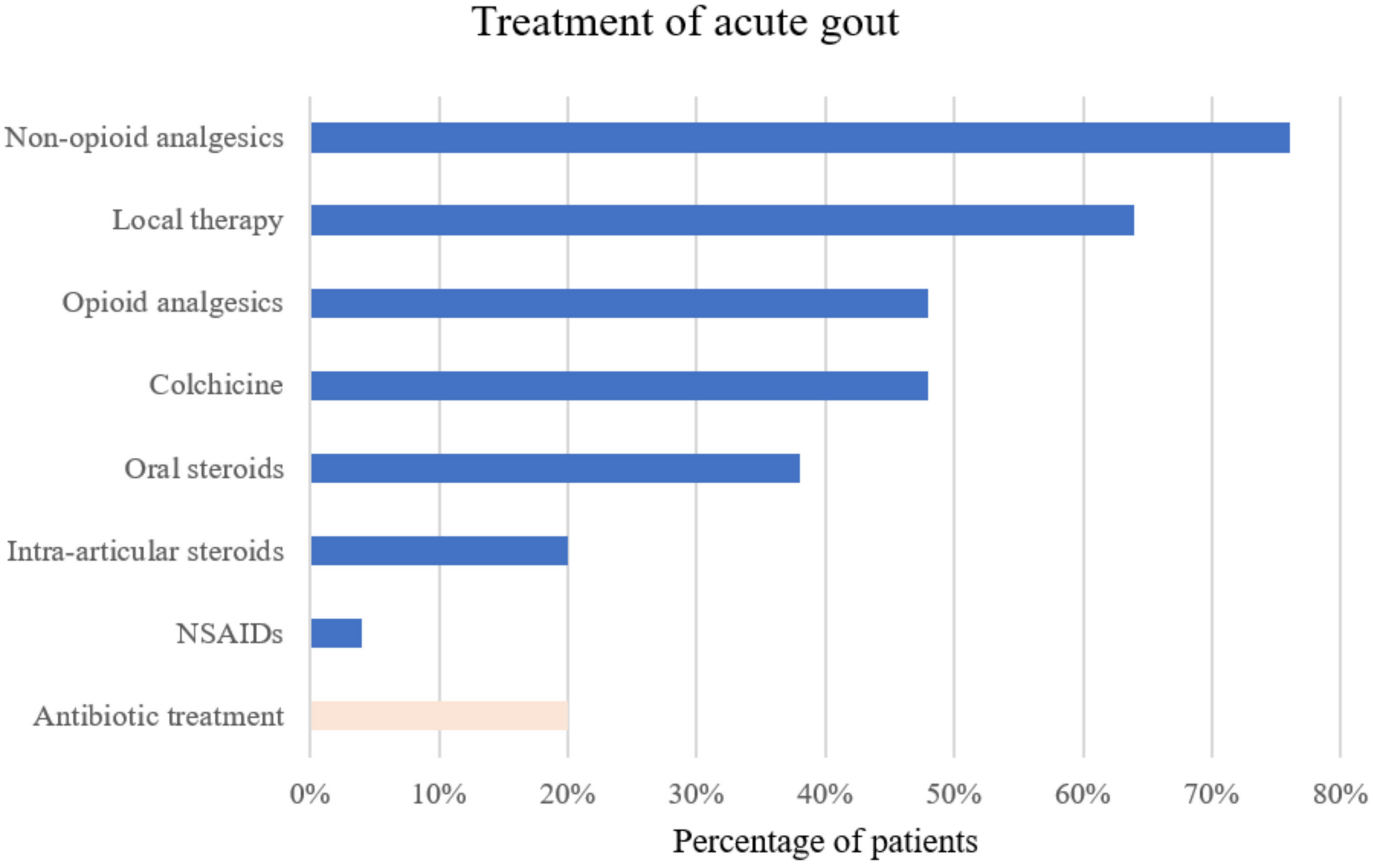
Treatment of the acute gout episode in percentage of patients receiving the therapy. NSAIDs, non-steroidal anti-inflammatory drugs.

For the treatment of the acute gout episode, the most commonly drugs used were colchicine in 24/50 (48%) and oral corticosteroids in 19/50 (38%) patients. Due to moderate to severe renal impairment, a reduced colchicine dose was often administered (i.e., initial dose of 1 mg followed by 0.5 mg 24 h later and continued once daily until flare resolution or during the period of optimization of urate-lowering therapy). Non-steroidal anti-inflammatory drugs (NSAIDs) were prescribed in 2/50 (4%) patients and intra-articular steroids injected in 10 (20%) patients. Non-opioid analgesics, opioid analgesics, and local therapy such as topical NSAIDs or cold packs were frequently used to alleviate the joint pain. A complete list of prescribed treatments around the acute gout episode is summarized in Figure 4. Interestingly, in 10 (20%) patients antibiotic treatment was started before a final diagnosis of an acute gout attack was established, underscoring the fact that infection was often entertained as a differential diagnosis.

Median peak temperatures during the gout attack [38.4°C (IQR 38.2–38.7) vs. 37.3°C (37.0–37.7), *p* < 0.0001] and CRP concentrations [179.7 mg/L (IQR 115.8 −221.3) vs. 77.8 mg/L (30.8–126.7), *p* = 0.001] were significantly higher in gDHF patients that received antibiotic treatment (*n* = 10) compared to gDHF patients without antibiotic treatment (*n* = 40). In line, joint aspiration and rheumatology consultation was performed in patients with a higher CPR (*p* = 0.005 for both comparisons). Interestingly, only two of the patients with a monoarticular arthritis pattern (10.5%) received antibiotic treatment compared to 25.8% of patients with polyarticular involvement (*p* = 0.28). Urate lowering therapy was newly initiated or adjusted after the gout attack in 20/50 (40%) of patients.

### Discharge Location and 6-Month Follow Up

In-hospital mortality was low (4% in both, gDHF and nDHF) in our cohort but only 36% of gDHF patients were directly discharged home after hospitalization. 23/50 patients were re-admitted within 6 months after discharge (median time to readmission 34 days). In 8 of them, the reason for readmission was due to a repeat heart failure exacerbation. At 6 months, 16/50 (32%) of patients had died. In contrast, nDHF patients were discharged home more frequently (58%) and had a markedly lower 6-month mortality 228/1,391 (16%).

## Discussion

The present in-depth analysis of DHF patients who developed an acute gout attack during hospitalization sheds light on factors, which are associated with an acute gout attack and may be important for the management of future heart failure exacerbations.

In our setting, almost 4% of patients admitted for DHF were diagnosed with an acute gout attack during hospitalization. This figure is in line with a case series of DHF patients from the U.S.A (11), but substantially lower compared to a cohort study of hospitalized patients in New Zealand with a history of gout who developed an acute gout flare (14%) (9). Assuming a similar prevalence in our entire heart failure cohort of comorbid gout (4.2%) as in a Swedish registry (16), it is tempting to speculate that a history of gout is the strongest risk factor for an acute gout attack during DHF admission. Indeed, the prevalence of comorbid gout was 70% in the present DHF patients that subsequently developed an acute gout attack.

Our analysis provides evidence that the prevalence of classical risk factors for gout, e.g., male gender, chronic kidney disease, and elevated serum uric acid levels is high at the time of admission in DHF patients with a subsequent inpatient gout attack. This is also underscored by the fact that these risk factors were much less prevalent in nDHF patients. Additional risk factors for an acute gout attack such as intensified diuretic use during hospitalization or discontinuation of urate lowering therapy were frequently observed and may have increased the risk of a potentially iatrogenic gout attack in this population. In the present analysis, we identified the characteristic DHF patient with a subsequent gout attack as being male, of advanced age, having several comorbidities including moderate acute or chronic renal failure, high uric acid concentrations and finally presenting with an oligo- or polyarticular arthritis. Our results mirror the risk factors identified by Jatuworapruk et al. in a cohort of gout patients developing an in-hospital gout flare (9). However, cardiovascular disease was the primary admission diagnosis in only 24% and diuretics adjusted in only 33% of patients that developed a gout attack during admission. Hence, it is unclear if the risk factors identified in their study are relevant for DHF patients.

Gout management was not optimal in the present gDHF study population. Despite a history of gout being present in most patients (70%), the majority of patients were not on ULT at the time of admission. On the other hand, for patients who were on ULT on admission, serum uric acid was not sufficiently lowered. A serum urate target below 360 μmol/l is recommended in gout management (15). This target was not reached in the vast majority (46/50, 92%) of our cohort on admission, indicating that a high serum uric acid level on admission in patients hospitalized for heart failure may be a key risk factor for inpatient gout attacks. Therefore, treat-to-target urate lowering therapy is crucial in this population. However, our analysis is limited by the fact that we did not evaluate uric acid concentration during steady state in these patients (i.e., before the decompensation). In addition, a selection bias toward poorly controlled patients is possible. Nevertheless, we are confident that the observed frequent target non-achievement is realistic, as a poor achievement of serum urate target during steady state was recently shown in a study with a multimorbid gout cohort from the index hospital (17).

In addition, we found that median serum uric acid concentrations increased during hospitalization in gDHF patients. As previously described, rapid changes in serum uric acid might lead to an acute gouty attack (18). Accordingly, every condition that causes alterations in extracellular urate concentration has the potential to trigger an attack. In our study, we observed several mechanisms, which might have triggered a change in serum urate concentration. First, higher doses of diuretics *per se* may lead to an elevation in serum uric acid concentration (due to uric acid retention) in a dose-dependent manner (19). Secondly, intensified diuretic regimens result in more aggressive diuresis and large volume status changes. As an adverse effect, intravascular volume and renal perfusion may be reduced, thereby increasing the risk of worsening renal function, which is reflected by a rise in creatinine and serum uric acid levels in our cohort. Additionally, in some patients, in-hospital adjustment (mostly cessation) of gout-related medications (probably as a consequence of an impaired renal function) may also have contributed to a change in urate concentration, thus promoting the development of an inpatient gout attack. Consequently, the awareness of all these factors may help to anticipate inpatient attacks in hospitalized heart failure patients who are at great risk for an acute gout attack.

In line with the newest ACR guideline recommendations (20) colchicine, corticosteroids or NSAIDs were primarily administered to treat the acute gout attack in our population. Not surprisingly, however, administration of NSAIDs was low in this heart failure population as current practice guidelines for heart failure therapy recommend to avoid NSAIDs (21). In addition, colchicine must be used cautiously in the setting of renal dysfunction as toxicity is increased in patients with chronic kidney disease (CKD). Similarly, corticosteroid treatment is not without risk in this population, given its potential to increase the bleeding risk and cause fluid retention or increased blood pressure. Despite the high prevalence of renal impairment the most commonly used drug in our cohort was colchicine, whereas corticosteroids were more frequently prescribed in another study (22). In a study of acute gout in hospitalized patients (19% of them suffering from congestive heart failure) by Petersel and Schlesinger (23), the frequency of chronic renal impairment was comparable to the present gDHF population and the drugs most commonly prescribed for acute gout in this population were colchicine (53%) and NSAIDs (51%). Different prescription patterns for treating gout probably reflect the above mentioned risks inherent to all gout attack drugs and that current guidelines do not further prioritize between these first-line agents in polymorbid patients. Intra-articular injections may offer a potential solution, that may be advantageous in the setting of a monoarticular gout attack and lack of anticoagulant treatment.

The finding of a high prevalence of comorbid conditions underscore the fact that gout management should be aligned with these frequent comorbidities. We therefore agree with Yoshida et al. (24) that management options that address several co-existing chronic conditions, including gout, would be ideal. Some medications originally designed for other indications have been demonstrated to increase urinary uric acid excretion and hence to lower serum uric acid levels. For example, losartan, an angiotensin receptor blocker, or fenofibrate, a lipid lowering drug, may show mutual benefits in the management of patients with gout and several other comorbidities (25, 26). Additionally, in a large observational study, sodium-glucose co-transporters-2 inhibitors (SGLT-2i), a medication recommended for patients with diabetes mellitus or heart failure with reduced ejection fraction, demonstrated a protective effect against gout attacks in patients with type 2 diabetes (27). In the present cohort, 19/50 patients had an ejection fraction <40% with a potential indication for a SGLT-2i.

Our analysis also illustrated that in some cases extensive work-up was performed to establish the diagnosis of an acute gout attack. The frequent consultation of rheumatologists underscores that arriving at a diagnosis of an acute gout attack in a hospital setting is not trivial. This might be due to the fact that symptoms of acute gout attacks may present with a more insidious onset and may be predominated or preceded by systemic features such as fever or elevated inflammatory markers rather than joint symptoms. These non-specific features might mimic bacterial infections or other inflammatory conditions. In particular, the characteristic inflammation of the first toe joint (podagra) was observed only occasionally. Consequently, this resulted in a number of unnecessary tests (such as blood cultures or X-rays) and antibiotic treatment (20% of patients, in particular in patients with fever and higher CRP).

Regarding the impact on health care resource utilization, we found the median length of stay for gDHF patients to be 16 days. This is significantly longer compared to DHF patients in different registries who had a median LOS of 4–11 days, and compared to nDHF patients in our hospital (9 days) (28). For example, the EuroHeart Failure Survey II (EHFS II) registry performed in Europe, reported a median LOS of 9 days (IQR 6–14) (29), which is identical to our nDHF cohort. Compared with nDHF patients, gDHF patients were more frequently male and suffering from more comorbidities including a more pronounced renal impairment. The high mortality rate after 6 months in gDHF patients (32%), which was higher than the 1-year mortality in the EHFS II population (20.5%) (30) and twice as high compared to nDHF patients in our hospital, illustrates the high disease severity in these patients. Together, these findings may explain a longer hospital stay. However, as shown recently in some studies, in-hospital gout flares may itself have increased the average hospital stay and increased healthcare costs (10, 31, 32).

A number of limitations are present in our study including the small number of gDHF patients analyzed. In addition, a considerable number of patients with an acute gout attack during admission had to be excluded due to refused general research consent. Due to the retrospective design of the study, data collection was limited to the documentation in hospital records, and primary care physician notes were not available. Hence, we cannot exclude that we have selected incompliant or poorly controlled gDHF patients. Furthermore, the retrospective identification of an acute gout attack was only possible if such an event was correctly recorded in the diagnosis list. Another potential error might be a wrong diagnosis of an acute gout attack, as the gold standard for the diagnosis is the identification of urate crystals in a joint aspirate. However, we tried to minimize this bias by applying the ACR/EULAR gout classification criteria. Regarding the follow-up period, readmissions were assessed only at the index hospital. For the nDHF cohort the diagnosis of DHF was not verified by chart review. Therefore, only some key characteristics of the entire nDHF cohort are reported, but an analysis of variables associated with a gout attack in DHF patients was not performed. Lastly, this is a single-centre study and therefore, results may not be generalizable to other hospitals and countries.

## Conclusion

Our descriptive analysis showed that male gender, chronic kidney disease, a previous history of gout and elevated serum uric acid levels (all of which are well-known risk factors of gout) are frequently present at the time of admission in DHF patients with a subsequent inpatient gout attack. Additionally, intensified diuresis during hospitalization with subsequent worsening of renal function or discontinuation of urate lowering therapy may further increase the risk of a potentially iatrogenic gout attack in this population.

Since gout attacks during hospitalization in DHF patients seem to be associated with increased health care resource utilization and poor prognostic outcomes, it is desirable to identify patients at high risk and potentially initiate preventive treatment strategies in selected patients. However, to identify risk factors of in-hospital gout attacks in DHF patients and correctly evaluate its impact on health care utilization, future case-control studies are required in this population to validate the present findings.

## Data Availability Statement

The original contributions presented in the study are included in the article/supplementary material, further inquiries can be directed to the corresponding author/s.

## Ethics Statement

The studies involving human participants were reviewed and approved by the Ethics Committee of Northwest and Central Switzerland (EKNZ Project-ID 2020-00547) with a waiver of informed consent for patients without a documented refusal of the general research consent of the University Hospital Basel.

## Author Contributions

FR and MO had full access to all of the data in the study, take responsibility for the integrity of the data, the accuracy of the data analysis, and prepared a first manuscript draft. FR collected the data. All authors contributed substantially to the study design and interpretation, analyzed the data, contributed substantially to the writing of the manuscript, critically revised the manuscript for important intellectual content, and approved the final version.

## Conflict of Interest

The authors declare that the research was conducted in the absence of any commercial or financial relationships that could be construed as a potential conflict of interest.

## Publisher's Note

All claims expressed in this article are solely those of the authors and do not necessarily represent those of their affiliated organizations, or those of the publisher, the editors and the reviewers. Any product that may be evaluated in this article, or claim that may be made by its manufacturer, is not guaranteed or endorsed by the publisher.
